# Physical activity as treatment for alcohol use disorders (FitForChange): study protocol for a randomized controlled trial

**DOI:** 10.1186/s13063-017-2435-0

**Published:** 2018-02-14

**Authors:** Mats Hallgren, Victoria Andersson, Örjan Ekblom, Sven Andréasson

**Affiliations:** 1 Department of Public Health Sciences, Karolinska Institutet, Solna, 171 77 Sweden; 2 Center for Psychiatric Research, Stockholm, Sweden; 3 Swedish School for Sport and Health Sciences, Stockholm, Sweden

**Keywords:** Alcohol dependence, Randomized controlled trial (RCT), Exercise, Physical activity, Yoga

## Abstract

**Background:**

Help-seeking for alcohol use disorders (AUDs) is low and traditional treatments are often perceived as stigmatizing. Physical activity has positive effects on mental and physical health which could benefit this population. We propose to compare the effects of aerobic training, yoga, and usual care for AUDs in physically inactive Swedish adults.

**Methods:**

This is a three-group, parallel, single-blind, randomized controlled trial (RCT). In total, 210 adults (aged 18–75 years) diagnosed with an AUD will be invited to participate in a 12-week intervention. The primary study outcome is alcohol consumption measure by the Timeline Follow-back method and the Alcohol Use Disorders Identification Test (AUDIT). Secondary outcomes include: depression, anxiety, perceived stress, sleep quality, physical activity levels, fitness, self-efficacy, health-related quality of life, and cognition. Blood samples will be taken to objectively assess heavy drinking, and saliva to measure cortisol. Acute effects of exercise on the urge to drink alcohol, mood, and anxiety will also be assessed.

**Discussion:**

The treatment potential for exercise in AUDs is substantial as many individuals with the disorder are physically inactive and have comorbid health problems. The study is the first to assess the effects of physical activity as a stand-alone treatment for AUDs. Considerable attention will be given to optimizing exercise adherence. Both the feasibility and treatment effects of exercise interventions in AUDs will be discussed. The Ethical Review Board (EPN) at Karolinska Institutet has approved the study (DNR: 2017/1380-3).

**Trial registration:**

German Clinical Trials Register, ID: DRKS00012311. Registered on 26 September 2017.

**Electronic supplementary material:**

The online version of this article (doi:10.1186/s13063-017-2435-0) contains supplementary material, which is available to authorized users.

## Background

Alcohol use disorder (AUD) is a debilitating medical condition associated with negative health and social outcomes. Key features include an inability to control the amount of alcohol consumed, consumption that results in social or work-related problems, and tolerance or the need to drink increasing amounts of alcohol to obtain a desired effect [1]. Rates of help-seeking for AUDs are typically low and traditional treatments, such as medication and counseling, are often perceived as stigmatizing [2]. Treatment outcomes are also highly variable with many patients relapsing into the dependence syndrome following a period of abstinence [3]. Available evidence suggests that many people with an AUD are insufficiently active [4] and have impaired cardiorespiratory fitness [5]; factors contributing to a higher prevalence of cardiovascular disease, diabetes, and metabolic syndrome [6, 7]. Of particular concern, even individuals with milder forms of AUD experience an excess mortality rate twice as high than those without the disorder [8].

Physical exercise is known to benefit mental and physical health synergistically [9], and may be effective in the treatment of AUDs where multimorbidity – the presence of two or more health problems – is common. A growing body of evidence suggests that individuals with substance use disorders are interested in exercising and may derive benefits from regular exercise, both in terms of general health/fitness and dependence recovery [10]. Cross-sectional and prospective studies have consistently shown that physical activity is associated with better mental health [11, 12], and recent studies indicate that exercise may help reduce tobacco cravings and cigarette use [13].

The effects of exercise on alcohol consumption remain understudied and poorly understood. A recent meta-analysis identified 21 studies published between 1972 and 2016 examining physical activity interventions for AUDs [14]. The results indicated positive effects of exercise on depression and physical fitness, but no significant changes on measures of consumption; however, all analyses for this outcome were limited to three studies. Considerable study heterogeneity was observed, and no interventions had examined the effects of exercise alone with traditional treatments for AUDs [14]. In a recent narrative review, Giesen et al. concluded that exercise is both safe and feasible for patients with alcohol dependence, but called for larger controlled trials with comparisons of different types of exercise [10].

One issue that remains unclear is how different types of exercise influence treatment response. In mental health research, both aerobic exercise and strength training are effective in the treatment of mood disorders [15, 16], with effect sizes comparable to medication and psychological therapy [16]. However, these interventions may not be suitable for all patients with an AUD, especially those with little or no exercise experience. With its focus on flexibility, balance and posture, yoga is an increasingly popular form of exercise that could be particularly suitable for those with an AUD. Yoga meets the formal definition of “physical exercise” because it is purposeful, repetitive, and engaged in to improve fitness and/or health [17]. However, most forms of yoga include additional core elements, such as mindfulness, a focus on controlled breathing, and relaxation techniques. These features are known to have an influence on depressive symptoms and wellbeing [18]. Moreover, previous studies found significant heterogeneity in trials incorporating these mind-body approaches when compared with conventional aerobic or strength exercises [19]. For these reasons, it is important to compare the effects of yoga with conventional aerobic training.

This project builds on recently published data demonstrating the feasibility of yoga as an adjunct treatment for alcohol dependence [20]. Extending this work, we will conduct a randomized controlled trial (RCT) to examine the effects of two 12-week, supported physical activity interventions (yoga and aerobic training) on alcohol consumption in physically inactive adults with an AUD. A comparison group will receive standard treatment, described below.

### Key research questions

- What are the effects of yoga and aerobic exercise on alcohol consumption in AUD patients?
- Does regular exercise reduce depression and anxiety, and improve self-efficacy in those with an AUD?
- Does exercise reduce stress and improve sleep quality in AUDs?
- Can exercise improve cognitive functioning in AUD patients?
- Does regular exercise improve health-related quality of life in AUD patients?
- What are the effects of exercise on cardiovascular risk factors and sedentary behavior?
- What are the effects of acute exercise on the urge to drink alcohol, mood, and anxiety? (nested study)
- What are participants’ subjective experiences of the intervention? (qualitative study)

## Methods

**Fig. 1:**
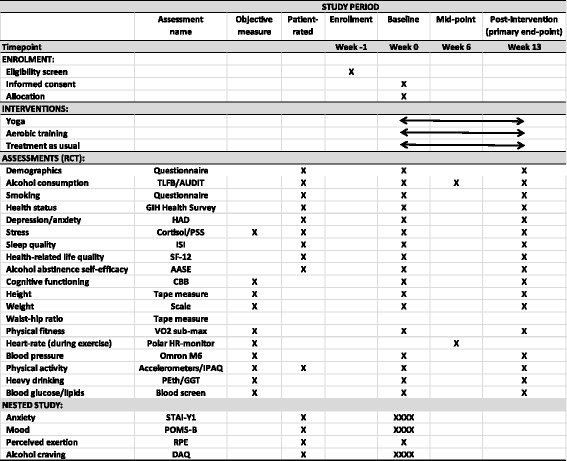
Standard Protocol Items: Recommendations for Interventional Trials (SPIRIT) Figure. For the acute (nested) study, assessments will be administered on four occasions at baseline (indicated by XXXX): 1 h before, immediately before, 5 min after, and 1 h after the fitness test. See description within the manuscript for more details

We adhered to the Standard Protocol Items: Recommendations for Interventional Trials (SPIRIT) guidelines in the preparation of this protocol (see Fig. 1 and Additional file 1 for the SPIRIT Figure and Checklist, respectively)

### Setting and participants

Project coordination will take place at “Riddargatan 1: Center for Alcohol and Health”; an outpatient treatment clinic located in central Stockholm specializing in AUDs. The clinic opened in 2011 and is staffed by physicians, psychologists, and allied health workers with expertise in the treatment and management of addictive behaviors, including AUDs. All participants will have a diagnosed AUD, based on *Diagnostic and Statistical Manual of Mental Disorders, version 5* (DSM-5) criteria. In addition to this criteria, participants must also meet the threshold for hazardous drinking, which is defined by the Swedish National Institute for Public Health as either (a) consumption of 9 or 14 standard drinks per week for women and men, respectively, or (b) binge drinking at least once during the past month (that is >4/5 standard drinks for men and women, consumed on a single occasion). Recruitment will occur through advertisements placed in waiting rooms in primary and occupational healthcare centers in Stockholm, and media outlets, e.g., local newspapers. Participants will not be recruited directly from the specialist outpatient clinic (Riddargatan 1) because these are individuals already seeking and expecting to receive specialized treatment. Recruitment will commence in January 2018 and continue throughout the year. The study will conclude in December 2019.

### Study design, randomization, and blinding

**Fig. 2:**
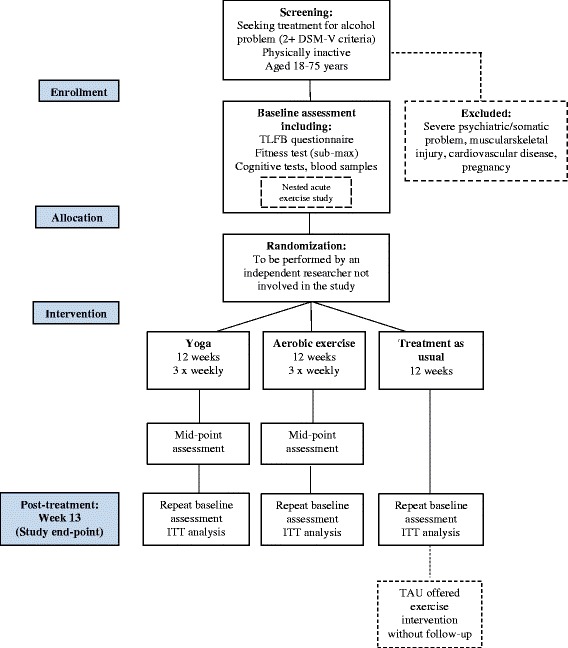
Participant flow diagram

This is a three-group, parallel, single-blind, RCT to compare the effects of three 12-week interventions (yoga, aerobic training, and treatment as usual (TAU)) on alcohol consumption. The proposed flow of participants through the trial is shown in Fig. 2. Assessments will be made at baseline (pre randomization) and post treatment (week 13). The randomization sequence will be computer generated and performed externally by a statistician at the Karolinska Institutet. A researcher not involved in the trial, but located at the clinic where the study will be managed, will then receive the allocation in sequentially numbered, opaque envelopes. In this way, the allocation sequence will be concealed from the person responsible for randomizing study participants. Trained research assistants, including a qualified nurse or psychologist, will administer each assessment in person at Riddargatan 1. The 12-week “treatment” will commence approximately 1 week after the baseline assessment when participants begin training/treatment (baseline physical activity levels will be measured for 1 week before this, see description below). To optimize recruitment and minimize dropouts, those randomized to TAU will be offered the 12-week exercise intervention after they have completed the follow-up assessment. The study endpoint is week 13 (post-treatment). All participants will be aged 18 to 75 years and residents of Stockholm County. Treatment allocation will not be concealed from either participants or the researchers conducting the baseline assessments. However, as a different research assistant will perform the post-treatment assessments, follow-up assessors will be blind to group allocation. Unblinding will only occur in the event that a participant chooses to withdraw from the study.

### Procedure

Prior to starting the RCT, a pilot study (*n* = 15) will be conducted involving an intervention identical to that used in the main study. The purpose will be to test the study protocol and obtain feedback from participants on the feasibility of the interventions. Interested participants will be advised to contact the project coordinator (a qualified nurse and clinician) for information about the trial. An initial telephone-based screening interview will be performed by the coordinator to determine eligibility based on age, health status, exercise participation history, and alcohol use. Those eligible will be invited to attend the clinic, where the purpose of the study will be explained in detail – verbally and in writing – and a baseline assessment completed after obtaining informed consent.

#### Exclusion criteria

Patients with known cardiovascular risks (e.g., a history of heart disease), hypertension (e.g., systolic blood pressure > 200 mmHg and/or diastole blood pressure > 110 mmHg), those with musculoskeletal injuries preventing exercise, those undertaking current regular exercise participation (defined here as two or more planned exercise sessions per week during the past month), and those requiring or receiving specialist psychiatric care (e.g., for psychosis) or with a primary somatic complaint will be excluded. A general health questionnaire developed by the Swedish Institute of Sport and Health Sciences (GIH) will be used to assess cardiovascular and related health risks. Patients who are at suicide risk, assessed initially by a single question “have you recently had thoughts about harming yourself?” will also be excluded and referred to a clinician for support. After completing the baseline assessment (approximately 90 min – see below), eligible participants will be requested to provide a blood sample at a registered laboratory, and return two saliva samples (morning and evening) using a sampling kit provided to them. On the same day all participants will also complete a baseline fitness test, and will be fitted with accelerometers to objectively measure physical activity levels for 1 week before starting the intervention.

#### Baseline and follow-up assessments

- Alcohol consumption assessed by the Timeline Follow-back method (TLFB) [21] is the primary study outcome. Using a calendar, respondents provide retrospective estimates of their daily drinking over the previous month. The TLFB has been evaluated with clinical and non-clinical populations [22]
- The Alcohol Use Disorders Identification Test (AUDIT): a 10-item screening instrument to assess alcohol consumption, drinking behaviors, and harms caused by alcohol use [23]
- Demographics: age, gender, occupation, marital status (baseline only)
- General health survey (developed by the GIH) for the assessment of cardiovascular disease and musculoskeletal problems (baseline only). Examples of questions include: have you ever been treated for heart disease? Do you have any injuries that prevent or interfere with exercise?
- The International Physical Activity Questionnaire (IPAQ) [24]: seven questions assessing the frequency and amount of time spent doing light, moderate, vigorous physical activity, and time spent sitting during the past 7 days
- The Hospital Anxiety Depression Scale (HAD) [25]: 14 items scored 0–3 based on how the respondent felt during the past week. Examples: I feel tense or “wound up”; I get a sudden feelings of panic; I feel as if I am slowed down
- The Perceived Stress Scale (PSS) [26]: 10 items scored 0–4 based on how the respondent felt during the past month. Examples: how often have you felt nervous and “stressed”?; how often have you felt that you were on top of things?; how often have you been able to control irritations in your life?
- The Insomnia Severity Index (ISI) [27]: seven items scored 0–4 based on the respondent’s self-rated sleep quality during the past 2 weeks. Examples: difficulties falling asleep; difficulties staying asleep; problems waking up too early
- The SF-12 Health Survey [28]: 12 items assess functional health and wellbeing from the respondent’s perspective. The SF-12 covers eight domains: vitality, physical functioning, bodily pain, general health perceptions, physical role functioning, emotional role functioning, social role functioning, and mental health
- The Alcohol Abstinence Self-Efficacy Scale [29]: 12 items scored on a 5-point Likert scale assess how “tempted” or “confident” the respondent feels that they would be to drink in certain situations
- The Cogstate Brief Battery (CBB): a standardized test of cognitive functioning that measures four key domains: psychomotor function, attention, visual learning, and working memory [30, 31]. Participants complete the 12-min test on a laptop computer under supervision

#### Objective assessment of physical activity

In addition to the IPAQ self-report questionnaire, physical activity, and sedentary behavior will be objectively recorded using an accelerometer (ActiGraph GXT3). All participants, including TAU participants, will be instructed to wear the device for seven consecutive days 1 week before the first exercise class (baseline assessment of activity), 1 week after (week 13). Accelerometry is a non-invasive method of monitoring human rest/activity cycles, and the device will estimate gross motor activity during the intervention. This data will enable more sophisticated analyses of the associations between exercise, physical activity, sedentary behavior, and alcohol consumption to be explored. To obtain an objective intensity measure of the prescribed physical activity sessions, data on heart rate will be collected by providing Polar heart-rate monitors to participants randomized to yoga or aerobic training. Because it is not feasible to gather heart-rate data from all participants for the entire duration of the trial, the monitors will be worn for 1 week during the middle of the participant’s 12-week treatment, thereby providing an “average” indicator of activity intensity. These data will be collected by a research assistant who will meet the participants after 6 weeks.

#### Biomarkers

From blood samples, standard biomarkers of heavy drinking will be taken at each assessment: phosphatidyl ethanol (PEth), γ-glutamyl transferase (GGT). Changes in blood cortisol level (stress) will be assessed at each time point, plus blood lipid profiles and blood glucose as markers of health status.

#### Fitness and general health assessment

Fitness will be assessed pre and post intervention using a graded, sub-maximal exercise test to predict VO_2_max using the method described by Ekblom-Bak et al. [32]. In addition, body weight, height, waist and hip circumference, and blood pressure will be recorded. Participants will be instructed to avoid alcohol consumption for 24 h before the test. The rationale for assessing fitness is to determine whether the intervention leads to a measurable change in aerobic conditioning, and to explore possible associations between fitness and different study outcomes. The testing procedure will take place at Riddargatan 1, supervised by a qualified exercise physiologist.

### Acute exercise study

Alcohol “urges” – that is, a strong craving for alcohol – have been shown to predict relapse in dependent patients, and recent studies suggest that short bouts of exercise can reduce urges for tobacco in smokers [33, 34]. To examine the short-term effects of acute exercise on alcohol craving, mood, and anxiety in those with an AUD, participants will complete the eight-item Desire for Alcohol Questionnaire (Shortened) [35], the 30-item Profile of Mood States – Brief Form (POMS-B) [36], and the state subscale of the State-Trait Anxiety Inventory (STAI-Y1) [37] on four occasions: 1 h before the fitness test, immediately before, 5 min after, and 1 h after the test. During the final minute, Borg’s Rating of Perceived Exertion will be administered; a single-item Visual Analogue Scale, where respondents indicate how hard they feel they are currently exercising. The fitness test will take about 15 min to complete. For feasibility reasons, this will be a single-arm, acute exercise study, where within-group changes over time are examined.

#### Exercise intervention

All physical activity classes include between 5 and 40 people. Participants will be requested to attend three exercise sessions per week for 12 weeks. To maximize adherence and reduce dropout, participants within each exercise group will be given a choice of either three yoga-based classes, or three aerobic classes of comparable intensity and duration. One advantage of this approach, is that participants will have a greater selection of times and locations for their training. Removing this choice could make the intervention impractical for some participants and reduce adherence. All sessions will be undertaken at SATS; a modern fitness center with multiple locations throughout Sweden. To ensure that classes are delivered in a consistent and safe manner, all trainers complete a course in personal training administered by “SAFE” (www.safe-education.se). The exercise options include: beginner to intermediate-level yoga sessions that coordinate breathing and movement in a dynamic flow between different yoga positions, and “Body balance” classes which combine conventional yoga with stretching and pilates movements. For aerobic training: “spinning” classes will be offered, consisting of aerobic exercise on stationary cycles performed in groups, “Energy” classes – choreographed aerobic training to music consisting of dynamic, whole-body movements designed to increase heart rate, and “Body-combat” classes, consisting of aerobic training based around martial arts movements (kicks, punches) and related training (sit-ups, push-ups, body-weight squats, etc.).

#### Adherence

Data on participation in all exercise sessions will be collected via the SATS electronic entry system which records the type of session attended. At 12 weeks, participants receiving the exercise intervention will be encouraged to continue the regimen but without follow-up or monitoring. In addition, participants will be offered three 45-min sessions (at weeks 1, 4, and 8, approximately) with a SATS personal trainer to monitor progress and help maintain motivation.

#### Treatment as usual (TAU)

Participants randomized to TAU will be asked to contact by phone an alcohol information service “Alcohol Help” (www.alkoholhjalpen.se). A qualified alcohol treatment specialist – nurse, psychologist or similar – will advise participants about the availability of different treatment options for their alcohol-related problem. These options include referral to a general medical practitioner, brief telephone counseling, referral to Alcoholics Anonymous or an Internet self-help website, or to a specialist alcohol treatment clinic. Participants may choose to pursue any (or none) or these treatment options. The treatment specialist will be given the participant’s AUDIT scores to facilitate feedback, and will contact each TAU participant on three occasions (once a month) to follow up their treatment progress. The researchers will have no influence over the treatment choices made by those randomized to TAU.

#### Follow-up assessments and qualitative study

All participants will be contacted by a research assistant at week 6, about half way through the intervention, and invited to attend a short meeting. The purpose will be to (1) hand out the heart-rate monitors with instructions for use and (2) to assess changes in alcohol consumption. Participants who indicate a 50% or more increase in the total volume of alcohol consumed during the past month assessed by the TLFB will be removed from the trial and offered additional clinical support. This assessment will be made by a research assistant with qualifications in nursing or psychology. Participants will be contacted again during week 13 and invited to attend a final follow-up interview. Those who decline will be invited to complete a phone interview or given a shortened baseline questionnaire to return via mail (TLFB, HAD, and physical activity only). Participants will be contacted using a combination of phone calls, emails, and text messaging up to six times over a 2-week period. In addition to collecting quantitative data, we propose to complement these data with a qualitative study. At the post-treatment assessment we propose to conduct semi-structured interviews with participants to determine which aspects of the study, including the exercise intervention, assisted them (or not) and why. During this process, we also propose to assess the acceptability of the interventions among men and women, and younger and older participants. Initial selection will be based on the order of inclusion into the trial. Since we aim for an even distribution across treatment groups, age, and gender, this will also be considered in the selection process. This is a purposive sample; interviews will be analyzed continuously during the data collection period, and the exact number of participants will be determined when “saturation” is reached and no new themes emerge from the interviews. The qualitative part of the study, i.e., developing the interview guide, selecting the participants, data collection, and analyzing the interviews, will be supervised by an experienced qualitative researcher.

### Statistical analyses and power calculation

Baseline participant characteristics stratified by treatment group will be presented. Group differences will be tested using *t* tests for continuous variables and chi-square tests for categorical outcomes. Parametric test assumptions will be examined and reported. Non-parametric data will be transformed or alternatively analyzed using non-parametric statistical tests. Multiple imputation will be used to replace missing internal values where appropriate. Within-group changes in the primary outcome (alcohol consumption) and secondary outcomes (depression, anxiety, cognitive ability, etc.) will be analyzed using paired-sample *t* tests. To address the primary research question, intention-to-treat analyses will be performed. The effect of group allocation on alcohol consumption and all secondary outcomes at 3-month follow-up will be assessed using mixed linear regression models (logistic for binary outcomes), using both the mean difference (baseline to 3 months) and total scores as dependent variables. Where the total score is used as the outcome, baseline group differences in covariates will be adjusted. As adherence may be sub-optimal, per-protocol analyses will also be presented. Data from individual accelerometers will be pooled to objectively assess changes in physical activity levels before and after treatment. We propose to conduct sub-group analyses based on participant gender and age to assess the effects of these variables on alcohol consumption. All analyses will be performed using SPSS 22.0. All tests will be two-sided and *P* values of less than 0.05 considered statistically significant.

Our sample size calculation is based on a preliminary study of the effects of exercise on alcohol consumption by Brown et al., 2015 [38]. Using this study as a guide, we anticipate a standardized mean difference (effect size) of around 0.3 favoring the yoga and aerobic exercise groups equally, compared to TAU. Our recent meta-analysis identified a pooled dropout rate of 40% from exercise for AUD studies. Due to the incentives in our trial, we estimate a slightly lower dropout of around 30%. Based on these parameters, with an 80% power, two-tailed significance of 2.5% to allow for two primary between-group comparisons (yoga versus TAU, and aerobic exercise versus TAU), and 1:1:1 allocation, we estimate that a total sample size of 210 participants will be required (i.e., 70 in each group). We used the program G*Power to determine sample sizes [39].

## Discussion

The alcohol treatment clinic “Riddargatan 1” is currently trialing new treatment options for AUDs with an understanding that alcohol misuse is part of a spectrum of lifestyle imbalance; a notion that resonates well with many patients [3]. Viewed this way, changing one’s drinking behavior and taking up regular exercise is a natural progression towards a healthier lifestyle. An added advantage of this approach is that it avoids the “complete abstinence” message and it makes treatment a more attractive and less stigmatizing option for patients. This is important, as premature termination of treatment and non-adherence are factors consistently associated with worse patient outcomes, yet both are common problems in alcohol treatment regimes. A meta-analysis of alcohol treatment outcome studies found that average short-term abstinence rates were 21% for untreated individuals, compared to 43% for treated individuals, suggesting that current therapies are more effective than no treatment, but there is considerable opportunity for improvement [40, 41].

Also relevant is that around 75% of those diagnosed with an AUD report mild-to-moderate drinking problems (two to five DSM-5 criteria fullfilled), with a much smaller proportion having severe problems [42, 43]. A Swedish study found that the majority of people who met the DSM-IV criteria for alcohol dependence did not drink at the highest consumption levels, were not living alone, and were not unemployed [43]. Most of those surveyed had few diagnostic criteria fulfilled and few social problems. This large majority with “moderate” alcohol dependence are also an under-treated group that is less inclined to seek professional help [43]. Currently, the individuals ultimately treated in specialist clinics tend to have more severe drinking problems, and reach out for help as a last resort, in part due to the perceived stigma of specialist clinics and the treatments currently offered.

Recent studies show that people with AUDs are interested and willing to engage in exercise interventions [10]. We suggest that broadening the array of effective, non-stigmatizing treatment options for AUDs could increase help-seeking, particularly among the majority with less severe drinking problems, and potentially reduce treatment non-adherence and dropout. The high prevalence of comorbid somatic and psychiatric problems in this population is another area where prescribed physical exercise could be of value. Previous reviews indicate that exercise is feasible for those with AUDs and will likely have positive effects on physical fitness and depression [14]. A potential source of bias – one that exists in many RCTs – is that the trial may attract a disproportionate number of people with positive expectations about physical activity. Where possible, characteristics of those who decline to participate will be compared to the active participants. However, the results will be generalizable to adults seeking treatment for alcohol-related problems through primary healthcare settings.

Our meta-analysis of exercise interventions for AUD suggests that participant attrition (dropout) may be high. Indeed, non-compliance with new exercise regimens is common in the general population. To minimize the risk of treatment non-adherence, all participants will be offered three free sessions with a qualified personal trainer who will be instructed to help participants identify and overcome obstacles to regular exercise. In addition, participants will meet with a research assistant half way through the study (week 6) to give out heart-rate monitors, and to briefly discuss their progress. All exercise sessions will be monitored electronically, and those who do not attend any sessions for 1 week will be contacted by their assigned trainer and encouraged to continue. We provide all training facilities free of charge, and those who complete the post-treatment assessment will receive feedback on changes in their test scores (e.g., fitness and cognition).

This large community-based trial will add clinically relevant information about the effects of acute exercise on mood, anxiety, and alcohol craving, and the effects of two popular physical activities (yoga and aerobic training) on alcohol consumption and health-related outcomes. Of importance, the trial will be one of the first to explore the effects of physical activity as standalone treatment for AUD. Policy and treatment implications of the findings will be discussed in future papers.

## Additional file

Additional file 1: SPIRIT 2013 Checklist: recommended items to address in a clinical trial protocol and related documents. (DOC 120 kb)

## Abbreviations

AUD: Alcohol use disorder
Gymnastik- och idrottshögskolan – GIH: Swedish Institute of Sport and Health Science
RCT: Randomized controlled trial
Regionala Etikprovninsnämnden – EPN: Regional Ethics Committee
SPSS: Statistical Package for the Social Sciences

## References

[1] NIAAA. Alcohol Use Disorder: National Institute for Alcohol Abuse and Alcoholism (NIAAA); 2017. Available from: https://www.niaaa.nih.gov/alcohol-health/overview-alcohol-consumption/alcohol-use-disorders. Accessed 15 Aug 2017.

[2] Blomqvist J, Cunningham J, Wallander L, Collin I. Att förbättra sina dryckesvanoe – om olika mönster förändring och om vad vården betyder. En rapport från projektet “Läsningar på alkoholproblem”. [Improving drinking habits – different patterns of change and the importance of treatment] Socialtjänsten i Stockholm : Forsknings- och utvecklingsenheten. FoU-rapport 2007:5. (SoRAD Rapportserie, nr 42). Stockholm,Sweden.

[3] Andreasson S, Danielsson AK, Wallhed-Finn S (2013). Preferences regarding treatment for alcohol problems. Alcohol Alcohol.

[4] Smothers B, Bertolucci D (2001). Alcohol consumption and health-promoting behavior in a US household sample: leisure-time physical activity. J Stud Alcohol Drugs.

[5] Herbsleb M, Schulz S, Ostermann S, Donath L, Eisentrager D, Puta C (2013). The relation of autonomic function to physical fitness in patients suffering from alcohol dependence. Drug Alcohol Depend.

[6] Vancampfort D, Mugisha J, Hallgren M, De Hert M, Probst M, Monsieur D (2016). The prevalence of diabetes mellitus type 2 in people with alcohol use disorders: a systematic review and large scale meta-analysis. Psychiat Res.

[7] Vancampfort D, Hallgren M, Mugisha J, De Hert M, Probst M, Monsieur D (2016). The prevalence of metabolic syndrome in alcohol use disorders: a systematic review and meta-analysis. Alcohol Alcohol.

[8] Roerecke M, Rehm J (2013). Alcohol use disorders and mortality: a systematic review and meta-analysis. Addiction.

[9] Penedo FJ, Dahn JR (2005). Exercise and well-being: a review of mental and physical health benefits associated with physical activity. Curr Opin Psychiatry.

[10] Giesen ES, Deimel H, Bloch W (2015). Clinical exercise interventions in alcohol use disorders: a systematic review. J Subst Abuse Treat.

[11] Stubbs B, Koyanagi A, Schuch FB, Firth J, Rosenbaum S, Veronese N (2016). Physical activity and depression: a large cross-sectional, population-based study across 36 low- and middle-income countries. Acta Psychiatr Scand.

[12] Harvey SB, Overland S, Hatch SL, Wessely S, Mykletun A, Hotopf M. Exercise and the prevention of depression: results of the HUNT Cohort Study. Am J Psychiatry. 2018;175(1):28-36. 10.1176/appi.ajp.2017.16111223 28969440

[13] Roberts V, Maddison R, Simpson C, Bullen C, Prapavessis H (2012). The acute effects of exercise on cigarette cravings, withdrawal symptoms, affect, and smoking behaviour: systematic review update and meta-analysis. Psychopharmacology (Berl).

[14] Hallgren M, Vancampfort D, Giesen ES, Lundin A, Stubbs B. Exercise as treatment for alcohol use disorders: systematic review and meta-analysis. Br J Sports Med. 2017;51(14):1058-64. 10.1136/bjsports-2016-096814 28087569

[15] Martinsen EW, Hoffart A, Solberg O (1989). Comparing aerobic with nonaerobic forms of exercise in the treatment of clinical depression: a randomized trial. Compr Psychiatry.

[16] Cooney GM, Dwan K, Greig CA, Lawlor DA, Rimer J, Waugh FR (2013). Exercise for depression. Cochrane Database Syst Rev.

[17] Caspersen CJ, Powell KE, Christenson GM (1985). Physical activity, exercise, and physical fitness: definitions and distinctions for health-related research. Public Health Rep.

[18] Goyal M, Singh S, Sibinga EM, Gould NF, Rowland-Seymour A, Sharma R (2014). Meditation programs for psychological stress and well-being: a systematic review and meta-analysis. JAMA Intern Med.

[19] Bridle C, Spanjers K, Patel S, Atherton NM, Lamb SE (2012). Effect of exercise on depression severity in older people: systematic review and meta-analysis of randomised controlled trials. Br J Psychiatry.

[20] Hallgren M, Romberg K, Bakshi AS, Andreasson S (2014). Yoga as an adjunct treatment for alcohol dependence: a pilot study. Complement Ther Med.

[21] Cervantes EA, Miller WR, Tonigan JS (1994). Comparison of timeline follow-back and averaging methods for quantifying alcohol consumption in treatment research. Assessment.

[22] Sobell LC, Maisto SA, Sobell MB, Cooper AM (1979). Reliability of alcohol abusers’ self-reports of drinking behavior. Behav Res Ther.

[23] Babor TF, Higgins-Biddle JC, Saunders JB, Monteiro MG (2001). AUDIT: the alcohol use disorders identification test. Guidelines for use in primary care.

[24] Craig CL, Marshall AL, Sjostrom M, Bauman AE, Booth ML, Ainsworth BE (2003). International physical activity questionnaire: 12-country reliability and validity. Med Sci Sports Exerc.

[25] Zigmond AS, Snaith RP (1983). The hospital anxiety and depression scale. Acta Psychiatr Scand.

[26] Cohen S, Kamarck T, Mermelstein R (1983). A global measure of perceived stress. J Health Soc Behav.

[27] Buysse DJ, Reynolds CF, Monk TH, Berman SR, Kupfer DJ (1989). The Pittsburgh sleep quality index: a new instrument for psychiatric practice and research. Psychiatry Res.

[28] Ware J, Kosinski M, Keller SD (1996). A 12-item short-form health survey: construction of scales and preliminary tests of reliability and validity. Med Care.

[29] McKiernan P1, Cloud R, Patterson DA, Wolf Adelv Unegv Waya S, Golder S, Besel K. Development of a Brief AbstinenceSelf-Efficacy Measure. J Soc Work Pract Addict. 2011;11(3):245-53. Epub 2011 Aug 16. 10.1080/1533256X.2011.593445 PMC3614369 23559892

[30] Maruff P, Thomas E, Cysique L, Brew B, Collie A, Snyder P (2009). Validity of the CogState brief battery: relationship to standardized tests and sensitivity to cognitive impairment in mild traumatic brain injury, schizophrenia, and AIDS dementia complex. Arch Clin Neuropsychol.

[31] Cairney S, Clough A, Jaragba M, Maruff P (2007). Cognitive impairment in aboriginal people with heavy episodic patterns of alcohol use. Addiction.

[32] Ekblom-Bak E, Bjorkman F, Hellenius ML, Ekblom B. A new submaximal cycle ergometer test for prediction of VO(2max). Scand J Med Sci Sports. 2014;24(2):319-26. 10.1111/sms.12014 23126417

[33] Janse Van Rensburg K, Taylor A, Hodgson T, Benattayallah A (2009). Acute exercise modulates cigarette cravings and brain activation in response to smoking-related images: an fMRI study. Psychopharmacology (Berl).

[34] Janse Van Rensburg K, Taylor A, Benattayallah A, Hodgson T. The effects of exercise on cigarette cravings and brain activation in response to smoking-related images. Psychopharmacology (Berl). 2012;221(4):659-66. 10.1007/s00213-011-2610-z 22234380

[35] Khemiri L, Jayaram-Lindström N, Hammarberg A. Psychometric evaluation of a Swedish version of the Shortened Desires for Alcohol Questionnaire (Shortened-DAQ). J Subst Abuse Treat. 2017. 10.1016/j.jsat.2017.05.016 28673529

[36] McNair DM, Lorr M, Droppleman LF. Manual for the profile of mood states. San Diego:Educational and Industrial testing services. 1971.

[37] Speilberger CD, Gorsuch R, Lushene R, Vagg P, Jacobs G (1983). Manual for the state-trait anxiety inventory.

[38] Brown RA, Abrantes AM, Minami H, Read JP, Marcus BH, Jakicic JM (2014). A preliminary, randomized trial of aerobic exercise for alcohol dependence. J Subst Abuse Treat.

[39] Faul F, Erdfelder E, Buchner A, Lang A-G (2009). Statistical power analyses using G*Power 3.1: tests for correlation and regression analyses. Behav Res Methods.

[40] Monahan SC, Finney JW (1996). Explaining abstinence rates following treatment for alcohol abuse: a quantitative synthesis of patient, research design and treatment effects. Addiction.

[41] Moyer A, Finney JW (2002). Outcomes for untreated individuals involved in randomized trials of alcohol treatment. J Subst Abuse Treat.

[42] Grant BF, Goldstein RB, Saha TD, Chou SP, Jung J, Zhang HT (2015). Epidemiology of DSM-5 alcohol use disorder results from the national epidemiologic survey on alcohol and related conditions III. JAMA Psychiat.

[43] Andreasson S, Danielsson AK, Hallgren M (2013). Severity of alcohol dependence in the Swedish adult population: association with consumption and social factors. Alcohol.

